# Performance of next-generation sequencing for diagnosis of blood infections by *Klebsiella pneumoniae*

**DOI:** 10.3389/fcimb.2023.1278482

**Published:** 2023-12-01

**Authors:** Lei Wang, Na Liu, Lin Zhang, Likun Cui, Mengdie Zhu, Zhifang Li, Panpan Wang, Zhengbin Wang

**Affiliations:** Department of Emergency, The First Affiliated Hospital of Zhengzhou University, Zhengzhou, China

**Keywords:** *Klebsiella pneumoniae*, bloodstream infections, metagenomic next-generation sequencing, blood culture, other culture

## Abstract

**Objective:**

*Klebsiella pneumoniae* (Kp) bloodstream infections (BSI) can be a life-threatening opportunistic infection. We aimed to evaluate the diagnostic accuracy of metagenomic next-generation sequencing (mNGS) for Kp BSI.

**Methods:**

We retrospectively analyzed 72 patients suspected with bloodstream infection and mNGS Kp positive in peripheral blood, who were hospitalized in our hospital from January 2022 to January 2023. Clinical data and laboratory parameters were collected. All patients had blood drawn and other samples for blood mNGS, blood cultures (BC) and other cultures (OC). The accuracy of mNGS results was analyzed according to infection site, clinical indicators, therapeutic effect and routine culture results. The detection of pathogenic microorganisms by blood mNGS and routine culture was compared.

**Results:**

Among 72 infection patients, 29 cases (40.28%) were BC positive, 43 cases (59.72%) were other culture (OC) positive, 16 cases (22.22%) were both BC and OC positive, 56 cases were positive for both mNGS and routine culture. Among the 56 double-positive cases, mNGS and conventional cultures were completely consistent in 27 cases, partially consistent in 15 cases, and completely inconsistent in 14 cases. Using the clinical diagnosis as the reference standard, There were 51 cases consistent with the results of mNGS with Kp BSI, the clinical consistency was 70.83% (51/72). The coincidence rate of mNGS and clinical diagnosis was higher than that of BC (54.17%, 39/72), indicating a statistically significant difference between the two methods (*P*<0.01).

**Conclusions:**

Current evidence indicates that mNGS exhibits excellent accuracy for the diagnosis of Kp BSI. Although it cannot replace blood culture detection technology, it can be used as a supplement to provide stronger diagnostic capabilities for BSI and optimize treatment.

## 1 Introduction

*Klebsiella pneumoniae* (Kp) is a Gram-negative bacillus and an important opportunistic pathogen that causes infections of the respiratory tract, hepatobiliary system, and blood (Zhou et al., 2020), which are leading causes of morbidity and mortality worldwide (Larcher et al., 2023; Shapaka et al., 2023). Notably, Kp invasion of the bloodstream can lead to systematic infection in immunocompromised patients, especially those with liver and biliary system diseases (Liu P. L. et al., 2023). Bloodstream infection (BSI) refers to an acute infection of the blood by a pathogen that releases toxic substances, which can cause severe damage to tissues and organs (Goto and Al-Hasan, 2013; Ferrer et al., 2014; Martinez and Wolk, 2016). Blood culture (BC) is currently the gold standard for diagnosis of Kp BSIs (Maddalena et al., 2023).

Although BC is highly specific for diagnosis of BSIs, this method is time-consuming and greatly limited due to low positivity rates. Hence, alternative methods have been developed for detection of BSIs. Metagenomic next-generation sequencing (mNGS) is increasingly being applied for pathogen detection in clinical practice. The diagnostic value of mNGS was first reported in 2014 for a case of leptospirosis infection (Motro and Moran-Gilad, 2017; Gu et al., 2019; Cao et al., 2022; Nan et al., 2022; Liu Y. et al., 2023). The shotgun method can be used to detect a wide range of pathogens, including bacteria, viruses, fungi, and parasites, and is more suitable for detection of pathogens that cannot be identified with conventional protocols. Recent studies have compared the sensitivity and specificity of conventional culture methods and mNGS for diagnosis of BSIs. However, interpretation of the results of mNGS remains controversial for identification of pathogenic bacteria vs. the normal flora. Therefore, clarification of the agreement between mNGS and an actual clinical diagnosis can best reflect the clinical value of mNGS. Therefore, the aim of the present study was to evaluate and compare the diagnostic performance and clinical consistency of mNGS and conventional culture methods for BSIs with Kp.

## 2 Manuscript

### 2.1 Materials and methods

#### 2.1.1 Study population

**Figure 1:**
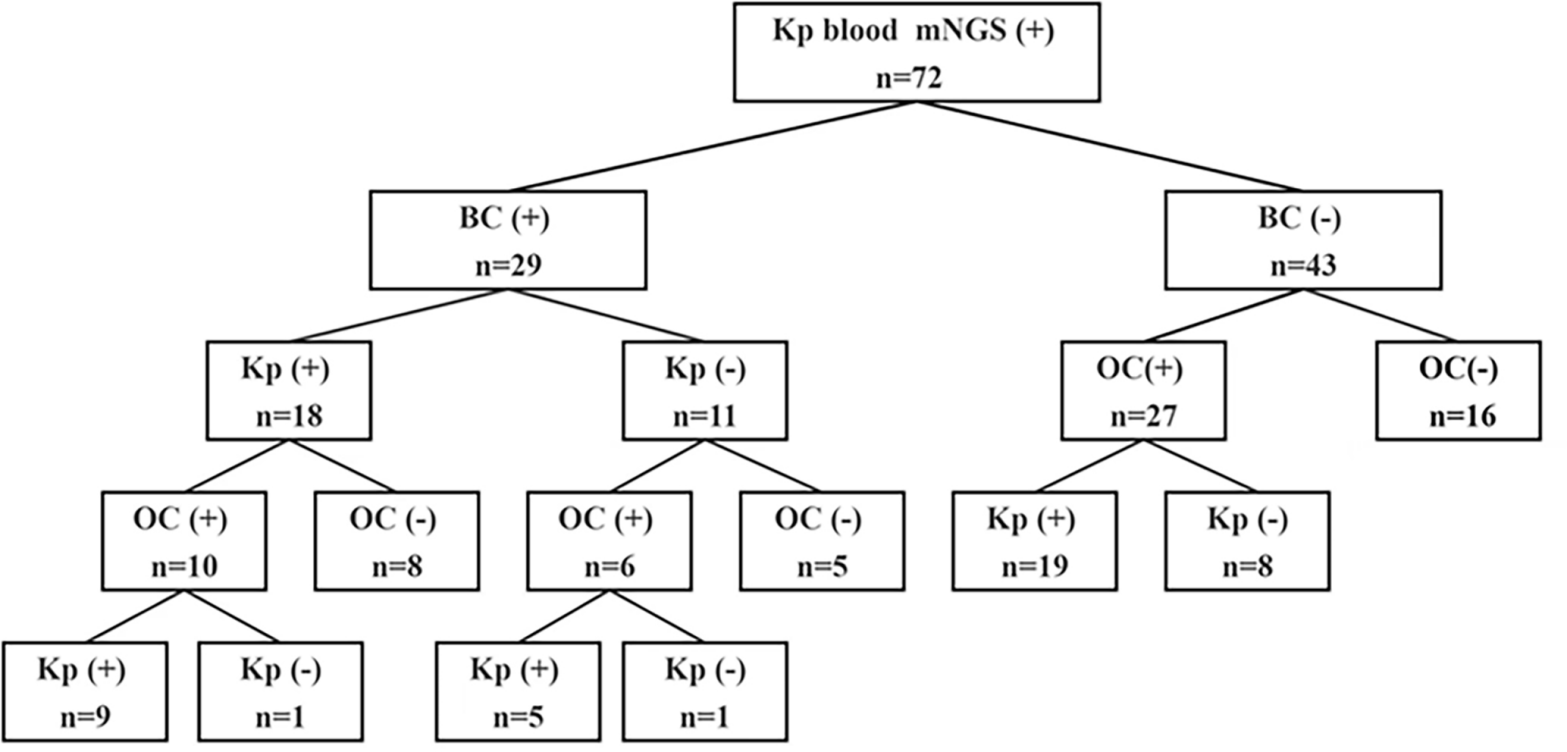
Flow chart of the study. Kp, *Klebsiella pneumoniae*; BC, blood culture; OC, other culture.

The cohort of this retrospective study included 72 patients with suspected Kp BSIs who were admitted to the First Affiliated Hospital of Zhengzhou University in Henan Province and underwent mNGS at Henan Provincial Gene Hospital from January 1, 2022 to January 1, 2023 (**Figure 1**). The study was limited to patients who met the following inclusion criteria: (i) suspected BSI with (ii) body temperature ≥ 38.0°C, (iii) C-reactive protein (CRP) ≥20 mg/L or procalcitonin (PCT) ≥0.5 ng/ml or hemodynamic instability, (iv) positive mNGS results in blood with Kp, and (v) coverage rate of 10-fold greater than any other microbe of the same type. Patients with incomplete clinical data, age <14 years, or lacking suitable specimens for mNGS were excluded from analysis. The clinical data of all patients were independently compiled by two doctors and approved by the chief physician. Age, sex, underlying diseases, serum levels of CRP, PCT, alanine transaminase (ALT), aspartate aminotransferase (AST), and creatinine (Cr), in addition to the type of BC and other indicators were collected from an Dongruan electronic medical record system (Neusoft medical, Shenyang, China). Furthermore, the collected data were anonymized and encrypted to protect patient privacy.

#### 2.1.2 Specimen collection and preparation

Within 24 hours of admission for blood culture, 20ml of whole blood samples (two bottles of aerobic and anaerobic) were collected using disposable sterile needles. Samples such as sputum, urine, drainage fluid, and alveolar lavage fluid were taken and submitted for analysis based on the requirements of their condition.

Peripheral blood samples (5 mL) were drawn into blood collection tubes, stored at room temperature for 3–5 min, and centrifuged (4,000 rpm, 10 min, 4°C). The plasma layer was transferred to a new sterile tube and microbial DNA was extracted using the QIAamp UCP Pathogen Kit (Qiagen GmbH, Hilden, Germany) in accordance with the manufacturer’s instructions for construction of DNA libraries.

#### 2.1.3 Bacterial culture

The BACT/ALERT 3D automatic bacterial culture instrument? was applied for testing the samples. At least two qualified medical professionals evaluated the positive results after the samples were incubated for 5 to 7 days in order to ensure the accuracy of the report. Once any blood culture produced positive results and environmental microbial contamination was ruled out, the final clinical positive result was determined.

#### 2.1.4 Construction of DNA libraries and sequencing

The extracted DNA was sonicated and DNA fragments of 200–300 bp were obtained. Then, DNA libraries were prepared using the TruePrep DNA Library Prep Kit V2 for Illumina^®^ (Vazyme Biotech Co., Ltd., Nanjing, China) in accordance with the manufacturer’s instructions. An Agilent 2100 Bioanalyzer (Agilent Technologies, Inc., Santa Clara, CA, USA) was used for quality control of the DNA libraries. Qualified libraries were pooled with other libraries using different index sequences and sequenced with a NextSeq 550Dx instrument (Illumina, Inc., San Diego, CA, USA) with the single-end 75-bp sequencing option.

#### 2.1.5 Quality control

In terms of reactor purity, we filtered 5 reactor boxes (3 columns, 2 magnetic beads), performed 2 biological replications, 3 technical repetitions. And different operators completed 200M high depth sequencing in 2 days, then selected the introduced reactor background bacterial species at least, and detected a relatively stable reactor box to carry out experiments. In terms of genome coverage controls, our sequencing target species are pathogens. First, we also used the above five reagent boxes to detect a mixture of 10 pathogenic bacteria (five positive bacteria, three negative bacteria and two fungi) to select the reagent box with the lowest extraction preference for different microorganisms. Second, in the course of the experiment, a host operation was taken to digest 90% human nucleic acid, reducing the proportion of humic nucleic acids in the sample, while each sample preset a total data volume of about 30M (the guidelines generally require >20M), with a view to achieving a higher genome coverage of the pathogen.

#### 2.1.6 Conventional cultivation and mNGS results read

A complete match was defined as positive results only for Kp infection by conventional culture methods. A partial match was defined as positive for Kp by conventional cultures, but also positive for other pathogens. A mismatch was defined as positive for other pathogens and negative for Kp by routine culture methods.

#### 2.1.7 Bioinformatics analysis

To generate high-quality sequencing data, low-quality reads were removed using Cutadapt v2.10 software (https://github.com/marcelm/cutadapt). Following removal of low-quality reads, the remaining data were classified by simultaneous alignment to the Pathogens Metagenomics Database, which consists of the sequences of 12895 bacteria, 1582 fungi, 11120 viruses, 312 parasites, 177 mycobacteria, and 184 mycoplasma/Chlamydia linked to human diseases. A customized Python script was used to identify species-specific full-length alignments with an identity of at least 95%. The remaining microorganisms were defined as credible if the following criteria were met: (i) the microbe had at least three non-redundant mapped reads per 10 million raw sequence reads (except for *Mycobacterium tuberculosis*); (ii) due to difficulty with detection, the sample was considered positive for the *M*. *tuberculosis* complex by identification of at least one taxon-specific, high-quality aligned read; and (ii) the microbe was potentially pathogenic.

#### 2.1.8 Statistical analysis

Continuous variables are expressed as the median and interquartile range (IQR, P25, P75), while categorical data are expressed as a number (percentage). Reference standards were used to evaluate the diagnostic efficacy of different indices. The diagnostic accuracy of mNGS and BC was evaluated and compared with the chi-square test. Data analyses were conducted using IBM SPSS Statistics for Windows, version 22.0. (IBM Corporation, Armonk, NY, USA) and MedCalc 18.11.3 software (https://www.medcalc.org/). A probability (*p*) value < 0.05 was considered statistically significant.

### 2.2 Results

#### 2.2.1 Patient characteristics

The characteristics of all patients with suspected Kp BSIs are summarized in **Table 1**. The study cohort included 72 patients (median age, 58 years; age range, 16–90 years) who were admitted to the First Affiliated Hospital of Zhengzhou University and underwent mNGS at Henan Provincial Gene Hospital from January 1, 2022 to January 1, 2023. Of these 72 patients, 57 (79.17%) were aged > 40 years and 48 (66.67%) were male. Details of underlying diseases and the laboratory test data are provided in **Table 1**. Among the 72 patients, 29 (40.28%) were positive for Kp BSIs by BC, 43 (59.72%) by other culture (OC) methods, and 16 (22.22%) by both BC and OC methods.

**Table 1:** Baseline characteristics of the participants.

Characteristic	Total(n=72)
Age, median (IQR)	58.50(46.50, 69.00)
≥40, n (%)	57(79.17)
<40, n (%)	15(20.83)
Gender, n (%)	
Male	48(66.67)
Female	24(33.33)
Alcohol, n (%)	22(30.56)
Smoking, n (%)	25(34.72)
Underlying disease, n (%)	
Hypertension	27(37.50)
Diabetes	21(29.17)
Cardiovascular disease	10(13.89)
Hemopathy	17(23.61)
Cerebrovascular disease	13(18.06)
Tumor(Excluding hematologic tumor)	10(13.89)
None	12(16.67)
Laboratory test, madian (IQR)	
PCT	2.69(0.47, 7.92)
CRP	145.43(35.55, 236.51)
ALT	24.00(13.00, 56.00)
AST	28.00(16.75, 49.25)
Cr	82.00(58.00, 149.00)
mNGS(+/-), n (%) BC(+/-), n (%) Other Culture(+/-), n (%)	72(100)/0(0) 29(40.28)/43(59.72) 43(59.72)/29(40.28)
BC and Other Culture(+/-), n (%)	16(22.22)/56(77.78)

IQR, interquartile range; PCT, Procalcitonin, CRP, C-reactive protein.

#### 2.2.2 Comparison of clinical diagnostic value and concordance between mNGS and conventional culture methods

**Figure 2:**
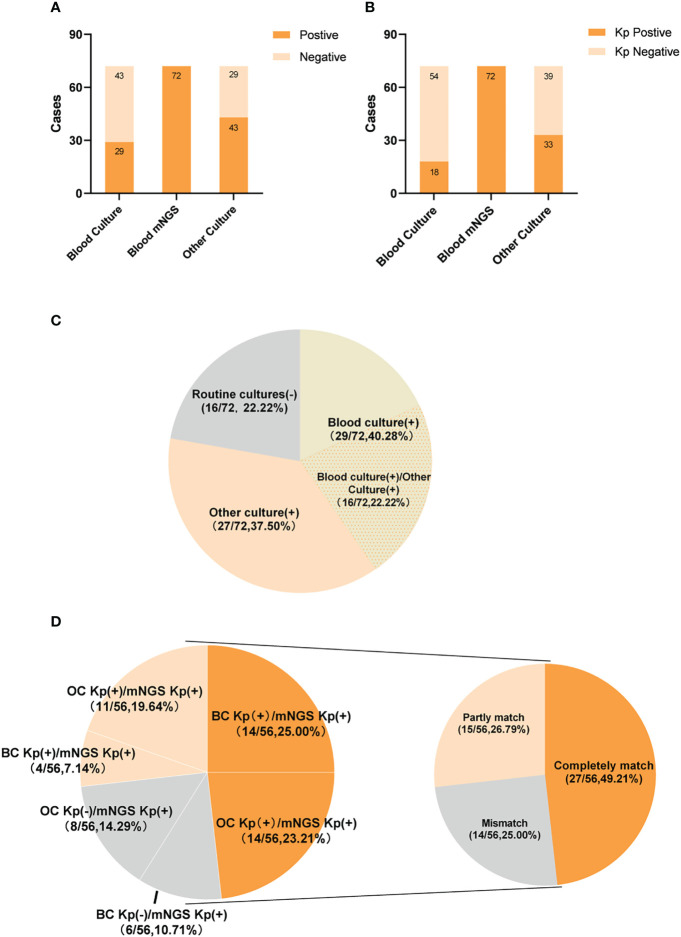
The positivity distribution between blood mNGS and conventional culture. **(A)** The pathogens positive cases of mNGS and conventional culture for all cases (n=72). **(B)** The Kp positive cases of mNGS and conventional culture for all cases (n=72). **(C)** Pie chart demonstrating the positivity distribution of mNGS and conventional culture for all cases(n=72). **(D)** Matching rate of *Klebsiella pneumoniae* infection in double-positive cases(n=56). The double-positive samples were further categorized as completely matched, partly matched and mismatched.

All 72 patients were Kp positive in blood samples by mNGS. Of these 72 patients, 29 (40.3%) were positive results by BC and 43 (59.7%) by OC methods (**Figure 2A**). In addition, 18 (25%) were positive for Kp infection by BC and 33 (45.8%) by OC methods (**Figure 2B**).

Of the 72 patients, 56 (77.8%) were positive by both mNGS and routine culture, while 16 (22.2%) were only positive by mNGS. Of these, 29 (40.3%) patients were positive by mNGS and BC, 27 (37.5%) by mNGS and OC methods, and 16 (22.2%) by mNGS, BC, and OC methods (**Figure 2C**). Among the 56 double-positive patient, the results of mNGS and conventional culture methods were in complete agreement for 27 (48.2%), partially consistent for 15 (20.8%), and completely inconsistent for 14 (19.4%) (**Figure 2D**).

#### 2.2.3 Positive samples by BC or OC methods

In total, 56 patients were positive by routine culture methods. Notably, the results of mNGS were completely inconsistent for 14 (25%) of the 56 patients with positive sample cultures. The peripheral blood specimens of six patients were positive for Kp infection by mNGS, while the BC results were positive for *Corynebacterium striatum* (case 31), *Staphylococcus haemolyticus* (case 44), *Staphylococcus aureus* (case 51), *Bacteroides fragilis* (case 58), *Escherichia coli* (case 65), and *Staphylococcus hominis* (case 69). Bronchoalveolar lavage fluid samples of five patients were positive for *Burkholderia cepacia* (case 20), *Candida albicans* and *Acinetobacter baumannii* (case 25), *Acinetobacter baumannii* (case 35), *Trichosporon* (case 39), or *Burkholderia cepacia* and *Stenotrophomonas maltophilia* (case 45), while the mNGS results were positive for Kp. Sputum and airway secretion samples of five patients were positive for *Staphylococcus aureus* (case 11), *Candida parapsilosis* (case 21), *Staphylococcus aureus*, *Acinetobacter baumannii*, and *Pseudomonas aeruginosa* (case 30); *Corynebacterium striatum* and *Acinetobacter baumannii* (case 31), or *Escherichia coli*, *Staphylococcus aureus*, and *Acinetobacter baumannii* (case 35); while mNGS of peripheral blood samples was positive for Kp. Furthermore, the cerebrospinal fluid of case 30 was positive for *Stenotrophomonas maltophilia*. The urine of case 35 was positive for *Candida tropicalis*, while mNGS was positive for Kp (**Table 2**).

**Table 2:** Samples with positive cultures.

Patient ID	mNGS	Blood culture	Sputum culture	BALF culture	Other culture
C01	*K. pneumoniae*, *P. aeruginosa*	Negative	*E. cloacae* complex group	*K. pneumoniae*, *P. aeruginosa*	Negative
C03	*K. pneumoniae*, HSV 5	*K. pneumoniae*, *E. faecium*	Negative	NA	*K. pneumoniae*, *E. faecium*, *S. hominis* subspecies (bile)
C04	*K. pneumoniae*	*K. pneumoniae*	NA	NA	NA
C05	*K. pneumoniae*	*K. pneumoniae*	NA	NA	NA
C06	*K. pneumoniae*, HSV 5	*K. pneumoniae*	NA	NA	*K. pneumoniae* (anal swab)
C 07	*K. pneumoniae*, JC polyoma virus	*S. haemolyticus*	NA	*K. pneumoniae*, *A. baumannii*	*C. albicans* (urine)
C 10	*K. pneumoniae*, Parvovirus type 9, HSV 4	*K. pneumoniae*	NA	NA	Negative
C 11	*K. pneumoniae*	Negative	S. aureus	NA	Negative
C 12	*K. pneumoniae*, *A. baumannii*, HSV1, HSV 4	*K. pneumoniae*, *E. faecium*	*K. pneumoniae*, *A. baumannii*	NA	*C. parapsilosis* (urine)
C 13	*K. pneumoniae*	Negative	*S. aureu*, aflatoxin	NA	*K. pneumoniae* (anal swab, airway secretion)
C 14	*K. pneumoniae*, *E. acteriaceae cloacae* complex, *P. mirabilis*	*A. baumannii*	*K. pneumoniae*, *E. hormaechei*	*A. baumannii*	Negative
C 16	*K. pneumoniae*, HSV5	Negative	*K. pneumoniae*, *S. maltophilia* strain	*C. glabrata*	*K. pneumoniae*, *S. maltophilia* strain (airway secretion); *S. maltophilia* strain (CSF); *C. glabrata* (urine)
C 17	*K. pneumoniae*, aflatoxin, HSV4	*K. pneumoniae*	NA	NA	NA
C19	*K. pneumoniae*, HSV 4	Negative	NA	*K. pneumoniae*	*K. pneumoniae* (airway secretion)
C20	*K. pneumoniae*, Parvovirus type 15, HSV5	Negative	NA	*B. cepacia*	*K. oxytoca* (anal swab), *E. faecium* (tip catheter culture)
C21	*K. pneumoniae*	Negative	NA	NA	*C. parapsilosis* (airway secretion)
C22	*K. pneumoniae*, *A. baumannii*, *P. aeruginosa*	Negative	*K. pneumoniae*	*A. baumannii*	*A. baumannii* (CSF)
C23	*K. pneumoniae*	*K. pneumoniae*	NA	NA	NA
C24	*K. pneumoniae*, *A. baumannii*, *B. cepacia*	Negative	Negative	Negative	*K. pneumoniae* (anal swab)
C25	*K. pneumoniae*, *S. anginosus*, *H. influenzae*	Negative	NA	*C. albicans*, *A. baumannii*	NA
C26	*K. pneumoniae*	Negative	NA	*K. pneumoniae*, *A. baumannii*	*A. baumannii*, *C. albicans* (urine)
C28	*K. pneumoniae*, *P. aeruginosa*, *A. baumannii*, *E. faecium*	Negative	NA	*K. pneumoniae*, *P. aeruginosa*, *A. baumannii*	*K. pneumoniae*, *A. baumannii* (airway secretion)
C29	*K. pneumoniae*, *C. tropicalis*	*K. pneumoniae*, *C. tropicalis*	Negative	NA	NA
C30	*K. pneumoniae*	Negative	Negative	NA	*S. aureus*, *A. baumannii*. *P. aeruginosa* (airway secretion) *S. maltophilia* strain (CSF)
C31	*K. pneumoniae*, *A. baumannii*, *E. faecium*, *Aflatoxin*, *A. fumigatus*	*C. striatum*	*A. fumigatus*	NA	*A. baumannii* (airway secretion)
C32	*K. pneumoniae*	*S. petenkofel*	*K. pneumoniae*, *P. aeruginosa*, *P. mirabilis*	*K. pneumoniae*, *P. aeruginosa*	*K. pneumoniae*, *P. aeruginosa* (airway secretion)
C35	*K. pneumoniae*, *E. coli*, Parvovirus type 29	Negative	*E. coli*, *S. aureus*	*A. baumannii*	*C. tropicalis* (urine), *E. coli*, *A. baumannii* (airway secretion)
C36	*K. pneumoniae*, *P. mirabilis*, Parvovirus type 29 HSV1, HSV 5, HSV 4	Negative	NA	NA	*K. pneumoniae*, *A. baumannii* (CSF)
C37	*K. pneumoniae*	Negative	*K. pneumoniae*	*K. pneumoniae*	Negative
C38	*K. pneumoniae*, *A. baumannii*, *C. striatum*, *S. aureus*	Negative	*A. baumannii*	NA	*K. pneumoniae* (urine)
C39	*K. pneumoniae*	Negative	NA	Trichosporon	NA
C40	*K. pneumoniae*, *A. baumannii*, *E. faecium*	*A. baumannii*	NA	NA	*K. pneumoniae* (airway secretion)
C41	*K. pneumoniae*	Negative	NA	NA	*K. pneumoniae* (airway secretion), *K. pneumoniae *(CSF)
C42	*K. pneumoniae*, *C. striatum*, *P. aeruginosa*, *S. Haemolyticus*, *A. baumannii*, *E. coli*	Negative	NA	*K. pneumoniae*, *B. cepacia*, *P. aeruginosa*, *A. baumannii*	*K. pneumoniae*, *P. aeruginosa*, *A. baumannii* (airway secretion)
C44	*K. pneumoniae*, HSV 4	*S. haemolyticus*	NA	NA	Negative
C45	*K. pneumoniae*, *A. baumannii*	Negative	NA	*B. cepacia*, *S. maltophilia* strain	*S. maltophilia* strain (airway secretion), *S. haemolyticus*, *C. neoformans* (CSF)
C46	*K. pneumoniae*	Negative	NA	*K. pneumoniae*	Negative
C47	*K. pneumoniae*, *P. aeruginosa*	*A. baumannii*	NA	*K. pneumoniae*, *A. baumannii*	*C. tropicalis* (urine)
C48	*K. pneumoniae*, *A. baumannii*,	*K. pneumoniae*, *E. faecium*	*A. baumannii*	*A. baumannii*, B. cepacia	NA
C50	*K. pneumoniae*, *E. coli*	*K. pneumoniae*	NA	NA	*K. pneumoniae* (pyogenic fluids)
C51	*K. pneumoniae*	*S. aureus*	NA	NA	Negative
C52	*K. pneumoniae*, HSV 5	*K. pneumoniae*	NA	NA	*K. pneumoniae* (airway secretion)
C53	*K. pneumoniae*, Parvovirus type 28, HSV 1, HSV 5	*K. pneumoniae*	NA	NA	NA
C54	*K. pneumoniae*	*K. pneumoniae*	*K. pneumoniae*, *K. aerogenes*	*K. pneumoniae*	NA
C57	*K. pneumoniae*	*K. pneumoniae*	NA	NA	*K. pneumoniae*, *E. faecium* (urine)
C58	*K. pneumoniae*, HSV 5	*B. fragilis*	NA	NA	NA
C59	*K. pneumoniae*	NA	NA	NA	*K. pneumoniae* (pyogenic fluids)
C60	*K. pneumoniae*, HSV1, HSV 4, HSV 5	*K. pneumoniae*	NA	NA	NA
C61	*K. pneumoniae*	NA	*P. aeruginosa*	NA	*K. pneumoniae* (anal swab)
C63	*K. pneumoniae*, *A. baumannii*	NA	*K. pneumoniae* *A. baumannii*	*A. baumannii*	NA
C64	*K. pneumoniae*, HSV 1, HSV 4	*K. pneumoniae*	*K. pneumoniae*	*K. pneumoniae*	NA
C65	*K. pneumoniae*, *E. coli*, *E. faecium*	*E. coli*	NA	NA	NA
C66	*K. pneumoniae*	NA	NA	NA	*K. aerogenes* (pyogenic fluids)
C67	*K. pneumoniae*, *B. fragilis*, *A. fumigatus*, HSV 5, Parvovirus type 6	*K. pneumoniae*	NA	NA	*K. pneumoniae* (ascitic fluid)
C69	*K. pneumoniae*	*S. hominis* subspecies	NA	NA	NA
C72	*K. pneumoniae*	NA	*K. pneumoniae*	NA	*K. pneumoniae* (pleural effusion, ascitic fluid)

NA, not available.

#### 2.2.4 Clinical diagnostic analysis

The three chief physicians make clinical diagnosis based on clinical symptoms, laboratory test results, routine culture results, and response to treatment. In the present study, the positive mNGS results were not in agreement with the BC results (six cases) and OC methods (eight cases). Of the six patients positive for Kp infection by BCs, none met the clinical criteria for BSI with Kp (positive BC result ruling out possible skin contamination), as most had severe underlying diseases and mixed infections with no evidence of Kp and poor or no response to broad-spectrum antibiotics (cases 31, 44, 51, 58, 65, and 69). Of the eight patients positive for Kp infection by OC methods, 4 (50%) met the criteria for Kp BSI, which included predilection sites, evidence of infection in medical records, response to antibiotic combination therapy, and significant improvement in markers of infection (cases 20, 21, 30, and 45). The other four patients did not meet the criteria and were considered negative for Kp BSIs (cases 11, 25, 35, and 39).

Of the 16 patients with negative results by conventional cultures, ten failed to meet the criteria of Kp BSIs. These ten patients had hematological malignancies with bone marrow suppression after chemotherapy and developed secondary infections. Of these ten patients, only five (50%) responded to broad-spectrum antibiotics and systemic antifungal drugs (cases 2, 18, 27, 33, and 50), while the other five patients (cases 8, 9, 34, 44, and 68) had died. On patient (case 71) had severe chronic diseases and no evidence of Kp infection. The remaining five patients (cases 15, 44, 49, 55, and 56) had determined as Kp infections and responded to antibiotics.

#### 2.2.5 Diagnostic performance of mNGS

The clinical diagnosis was consistent the mNGS results in 51 cases and the BC results in 39 cases. The accuracy of mNGS and clinical diagnosis was significantly greater than routine culture methods (70.83% vs. 54.17%, respectively, *p <*0.01) (**Table 3**), demonstrating that mNGS is superior to BC for diagnosis of BSIs.

**Table 3:** Diagnostic performance of mNGS for Klebsiella pneumoniae bloodstream infection.

	Consistency	Inconsistency	Accuracy	^2^	P-value
**mNGS**	51	21	70.83%	35.04	<0.01
**Blood culture**	39	33	54.17%		

### 2.3 Discussion

Kp is a threat to public health and can cause a range of severe infections, such as pneumonia, BSI, meningitis, urinary tract infection, and bacterial abscesses (Dong et al., 2019; Lai and Yu, 2021), which can result in high mortality rates of hospitalized patients (Shapaka et al., 2023). Based on virulence factors, Kp is commonly classified as classic or hypervirulent (Liu and Guo, 2018; Zhu et al., 2021; Lin et al., 2022). Routine culture methods for detection of Kp BSIs have relatively low positivity rates (10% to 30%) and are time-consuming (Zhou et al., 2023). As compared to traditional cultural methods, the benefits of mNGS include a high positivity rate, wide coverage, short turnaround time, and high throughput. mNGS is also unbiased because it analyzes all the genetic material that can be extracted from the sample nonspecifically (Li et al., 2022; Liu Y. et al., 2023; Wang et al., 2023). However, there is great controversy in the clinical interpretation of mNGS tests (Simner et al., 2018), and relatively few studies have investigated high positivity rate of mNGS and the clinical consistency.

In this study, peripheral blood samples of 72 patients with suspected Kp BSIs were assessed by mNGS. Then, the results of mNGS and traditional culture methods were compared to analyze the diagnostic performance of mNGS. Among these patients with suspected Kp BSIs, there were twice as many men than women and significantly more with underlying diseases than without. In addition, markers of inflammation, such as CRP and PCT, were significantly elevated in these patients. Serum levels of PCT and CRP rapidly increase in response to infection, thus these markers are commonly used to assess the severity of infection, particularly for Gram-negative bacilli (Cao et al., 2022).

Some of the patients in this study had simple Kp BSIs without underlying diseases, while others had secondary pulmonary or abdominal infections. Most patients in intensive care units (ICUs) had prior severe malignancies or hematologic diseases and developed myelosuppression after chemotherapy, secondary to Kp BSIs with other complex infections, which usually result in a poor prognosis. Among patients with hospital-acquired BSIs, 25% are positive at the time of admission to the ICU, while 75% were acquired during admission to the ICU. The incidence of ICU-acquired BSI is about 5%–7% (Bassetti et al., 2016; Adrie et al., 2017; Liu Q. L. et al., 2023). The EUROBACT-2 international cohort study found that ICU-acquired BSI was mainly caused by pulmonary infection (26.7%), catheter-associated infection (26.4%), and intraperitoneal infection (12%), while no primary site was identified in 24%. The most frequent pathogens of BSIs were Gram-negative bacteria (59.0%), predominantly *Klebsiella* (27.9%), *Acinetobacter* (20.3%), *Escherichia coli* (15.8%), and *Pseudomonas* (14.3%) (Tabah et al., 2023). Kp is the most common cause of BSIs in critically ill ICU patients.

In the present study, only 29 (40.3%) of 72 patients were positive by BC, which is a higher positivity rate than reported in previous studies (Hirosawa et al., 2023). As a possible explanation for the higher detection rate, the cohort of the present study was limited to patients with highly suspected BSIs and positive mNGS results. However, the detection rate was still lower for BC than mNGS (71%) after clinical analysis, consistent with previous reports (Long et al., 2016; Grumaz et al., 2019; Zhou et al., 2023). In addition to BC, OC methods are used for clinical diagnosis of many specimens of the respiratory tract, urine, cerebrospinal fluid, pus, and thoracoabdominal fluid, and those relatively sterile specimens have a higher evidence level for infection diagnosis (Li et al., 2023; Luo et al., 2023). OC methods do not provide direct evidence of BSI but can confirm sites of infection of pathogens that cause BSI, and the consistency of OC methods with mNGS provides evidence of BSIs. In this study, 43 (59.7%) of 72 patients were positive by OC methods. As a possible explanation for this high positivity rate, multiple specimens from the same patient were positive for infection with multiple microbes. For 9 patients, the results of BC, mNGS and OC methods were highly consistent. Although the results of OC methods for Kp infection were negative for one patient, the results of BC and mNGS were consistent for Kp BSI. The BC results were positive for other pathogens in 5 patients and negative for 19, while the results of OC methods and mNGS were highly consistent for Kp, indicating that positive results for OC methods provide indirect evidence of BSIs.

mNGS has shown great potential for diagnosis of infections of the blood, respiratory tract, and central nervous system. Many studies have demonstrated that mNGS is superior to conventional culture for diagnosis of infectious diseases. Overall, the positivity rate of mNGS for diagnosis of infectious diseases is 58.1%–82.1%, which decreases to 47.5% if viruses are excluded. Nonetheless, mNGS has a significantly higher positivity rate than culture (10%–30%). The high sensitivity of mNGS may be due to the relatively longer survival of pathogenic DNA and limited effect of antibiotics. Collectively, the results of this study further confirmed that the sensitivity of mNGS for diagnosis of infectious diseases is significantly higher than traditional etiological detection. Second, mNGS offers greater detection of a broad range of pathogens, which can compensate for the limitations of culture methods. It is widely accepted that mNGS is advantageous for the detection a broad range of pathogens, especially *Mycobacterium tuberculosis* in addition to various viruses, anaerobic bacteria, and fungi (Duan et al., 2021). In this study, mNGS detected other viruses and fungi in the blood samples of about 35% (25/72) of patients. More importantly, the results of mNGS were systematically analyzed and verified based on laboratory tests, auxiliary examination results, and clinical effects. In this study, whether from direct evidence of BSI, indirect evidence, or clinical analysis, the compliance rate of mNGS was higher than that of BC (71% vs. 54%, respectively), which may have been related to the low positivity rate of BC.

There were some limitations to this study that should be addressed. First, this was a retrospective study with a relatively small number of patients from a single institution, and there may be selective bias. We need to further expand the sample scope of research to enhance the reliability and spreadability of the results. Second, while the results of this study were based on clinical manifestations of patients combined with other laboratory results, subjective bias was greatly influenced by clinical experience. Third, the majority of microorganisms identified by mNGS were not verified by molecular tests. Finally, the majority of patients underwent treatment prior to analysis, which may have impacted the sensitivity of culture and mNGS.

In conclusion, the results of this study indicate that mNGS can precisely diagnose BSIs, but cannot completely replace BC. Hence, mNGS should be used as a supplementary method to provide stronger diagnostic capabilities for BSIs and optimize treatment.

## Data availability statement

The data presented in the study are deposited in the SRA database repository, accession number PRJNA1044068.

## Author contributions

LW: Conceptualization, Methodology, Writing – original draft. NL: Formal Analysis, Investigation, Writing – original draft. LZ: Formal Analysis, Investigation, Writing – review & editing. LC: Investigation, Writing – review & editing. MZ: Investigation, Writing – review & editing. ZL: Methodology, Writing – review & editing. PW: Methodology, Writing – review & editing. ZW: Writing – review & editing.
